# Acute vitamin C improves cardiac function, not exercise capacity, in adults with type 2 diabetes

**DOI:** 10.1186/s13098-018-0306-9

**Published:** 2018-02-14

**Authors:** Rebecca L. Scalzo, Timothy A. Bauer, Kylie Harrall, Kerrie Moreau, Cemal Ozemek, Leah Herlache, Shawna McMillin, Amy G. Huebschmann, Jennifer Dorosz, Jane E. B. Reusch, Judith G. Regensteiner

**Affiliations:** 1 Division of Endocrinology, Department of Medicine, University of Colorado School of Medicine (UCSOM), 12801 E17th Ave, Aurora, CO 80045 USA; 2 Division of General Internal Medicine, Department of Medicine, University of Colorado School of Medicine (UCSOM), Aurora, CO USA; 3 Division of Geriatrics, Department of Medicine, University of Colorado School of Medicine (UCSOM), Aurora, CO USA; 4 Division of Cardiology, Department of Medicine, University of Colorado School of Medicine (UCSOM), Aurora, CO USA; 5 Department of Medicine, Center for Women’s Health Research, University of Colorado School of Medicine (UCSOM), Aurora, CO USA; 6 Veterans Administration Medical Center (VAMC), Denver, CO 80215 USA; 7 VAMC-Geriatric Research Education and Clinical Center (GRECC), Denver, CO 80215 USA

**Keywords:** Cardiorespiratory fitness, Brachial artery flow mediated dilation, Oxygen uptake kinetics, Cardiac echocardiography

## Abstract

**Background:**

People with type 2 diabetes (T2D) have impaired exercise capacity, even in the absence of complications, which is predictive of their increased cardiovascular mortality. Cardiovascular dysfunction is one potential cause of this exercise defect. Acute infusion of vitamin C has been separately shown to improve diastolic and endothelial function in prior studies. We hypothesized that acute vitamin C infusion would improve exercise capacity and that these improvements would be associated with improved cardiovascular function.

**Methods:**

Adults with T2D (n = 31, 7 female, 24 male, body mass index (BMI): 31.5 ± 0.8 kg/m^2^) and BMI-similar healthy adults (n = 21, 11 female, 10 male, BMI: 30.4 ± 0.7 kg/m^2^) completed two randomly ordered visits: IV infusion of vitamin C (7.5 g) and a volume-matched saline infusion. During each visit peak oxygen uptake (VO_2_peak), brachial artery flow mediated dilation (FMD), reactive hyperemia (RH; plethysmography), and cardiac echocardiography were measured. General linear mixed models were utilized to assess the differences in all study variables.

**Results:**

Acute vitamin C infusion improved diastolic function, assessed by lateral and septal E:E’ (*P* < 0.01), but did not change RH (*P* = 0.92), or VO_2_peak (*P* = 0.33) in any participants.

**Conclusion:**

Acute vitamin C infusion improved diastolic function but did not change FMD, forearm reactive hyperemia, or peak exercise capacity. Future studies should further clarify the role of endothelial function as well as other possible physiological causes of exercise impairment in order to provide potential therapeutic targets.

*Trial registration* NCT00786019. Prospectively registered May 2008

## Background

People with type 2 diabetes (T2D) have impaired cardiorespiratory fitness [1–3] and increased cardiovascular mortality compared with non-diabetic controls [1]. The necessary integration of cardiovascular function as well as peripheral uptake and utilization of nutrients and oxygen during aerobic exercise makes cardiorespiratory fitness an indicator of whole-body physiological function and a powerful predictor of all-cause and cardiovascular mortality [3–6]. Improving cardiorespiratory fitness in people with T2D is one potential approach to address the risk for premature cardiovascular mortality in this population.

Our group has previously established that abnormal insulin sensitivity, endothelial dysfunction, and decreased cardiac perfusion and function, all of which are observed in T2D, are associated with reduced cardiorespiratory fitness [2–4, 7–9]. Moreover, evidence demonstrates that inappropriate skeletal muscle oxygenation in T2D may be associated with the exercise impairment in T2D [10–12]. Together, these data suggest that both central and peripheral components of physiological function are abnormal during exercise in T2D; however, the interrelationships of these factors and direct mechanisms causing the exercise impairments of T2D remain unclear.

Cardiovascular function is a primary contributor to exercise capacity and it is impaired in people with T2D, providing a logical potential cause of the impaired cardiorespiratory fitness of T2D. In previous studies, supplementation with antioxidants has augmented cardiac function [13–16] and vasodilation [17–19] which may impact tissue oxygen delivery in both central and peripheral circulations and affect the utilization of oxygen during exercise. However, to date we have not determined the isolated effects of cardiovascular function, per se, on cardiorespiratory fitness in persons with T2D. Therefore, the objective of this investigation was to evaluate the potential role of cardiovascular dysfunction as a unifying contributor to the exercise abnormalities observed in people with T2D. We hypothesized that acute vitamin C infusion would improve exercise capacity and that these improvements would be associated with improved indices of cardiovascular function.

## Methods

### Participants

Thirty-one sedentary adults (33–55 years old) with uncomplicated T2D and 21 sedentary healthy adults of similar body mass index were recruited and participated in the current investigation. Participant characteristics are presented in Table 1. The Institutional Review Board of the University of Colorado School of Medicine (UCSOM) approved the experimental protocol, and the nature, purpose and risks of the study were explained before written informed consent was obtained from each participant.

**Table 1:** Participant characteristics

	Control	T2D
n	21	31
Females (n)	11 (52.4%)	7 (22.6%)
Age (years)	45 ± 2	46 ± 1
Height (cm)	171 ± 2	174 ± 2
Body mass (kg)	89.2 ± 2.4	96.5 ± 3.3
Body mass index (kg/m^2^)	30.4 ± 0.7	31.5 ± 0.8
Fat-free mass (kg)	58.4 ± 2.3	64.6 ± 2.3
HbA-1c (%)	5.4 ± 0.1	6.9 ± 0.1*
Insulin (µU/ml)	15.1 ± 1.7	28.7 ± 3.4*
Glucose (mg/dl)	86.5 ± 1.4	121.2 ± 4.1*
Adiponectin (µg/ml)	9.5 ± 1.2	5.1 ± 0.4*
Total cholesterol (mg/dl)	173 ± 6	144 ± 4*
LDL cholesterol (mg/dl)	105 ± 5	85 ± 4*
HDL cholesterol (mg/dl)	52 ± 3	39 ± 1*
Triglycerides (mg/dl)	107 ± 8	143 ± 12*
Resting systolic blood pressure (mmHg)	121 ± 2	116 ± 2
Resting diastolic blood pressure (mmHg)	81 ± 2	78 ± 2

Data are mean ± SEM

* Different from control group (*P* < 0.05)

Presence of T2D was documented by chart review and presence of treatment for T2D. Persons with T2D were included if their diabetes was treated by diet alone, metformin and/or oral anti-diabetic medications and demonstrated adequate glycemic control with total HbA1C of 6.9 ± 0.1 on therapy. All participants were sedentary (defined as exercising one bout per week or less) and participation in the study was only permitted if participants did not plan to alter their exercise or diet efforts during the study. Women were tested during the mid-follicular phase of their menstrual cycle (7–10 days after the onset of menstruation) determined by serum follicle stimulating hormone or during days 5–7 of the placebo week if taking oral contraceptives.

History, physical examination and laboratory testing confirmed absence of comorbid conditions. Exclusion criteria included: cigarette use within 1 year prior to study, evidence of acute liver disease, evidence of distal symmetrical neuropathy [by evaluation of symptoms (numbness, paresthesia) and signs (elicited by vibration, pinprick, light touch, ankle jerks)], autonomic dysfunction (> 20 mmHg fall in upright BP without a change in heart rate), proteinuria (urine protein > 200 mg/dl) or creatinine ≥ 2 mg/dl, evidence of heart disease by history, echocardiography, or abnormal resting or exercise electrocardiogram (≥ 1 mm ST segment depression), angina or other cardiac or pulmonary symptoms potentially limiting exercise performance. Systolic blood pressure > 190 mmHg at rest or > 250 mmHg with exercise or diastolic pressure > 95 mmHg at rest or > 105 mmHg with exercise were also grounds for exclusion. All exclusions were made for reasons of participant safety or potential effects on exercise performance.

### Study protocol

**Fig. 1:**
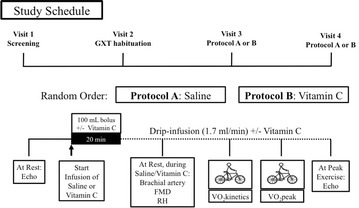
Study protocol overview

All visits occurred at the Regensteiner Vascular Research Laboratory of the Colorado Clinical Research Center at the UCSOM; a schematic representative of the study protocol is depicted in Fig. 1. During visit one, a history and physical examination was completed as well as a fasted venous blood drawn to determine circulating metabolic factors including glucose, insulin, cholesterol, and triglycerides. Sedentary lifestyle was confirmed using the low-level physical activity recall survey (LoPAR) [20]. In addition, a resting electrocardiogram and urine analysis were performed. During visit two, participants completed a graded cycle exercise test for habituation purposes and body composition was assessed via dual-energy X-ray absorptiometry (DEXA). Next, participants completed two randomly ordered visits: IV infusion of vitamin C (7.5 g) and a volume-matched saline infusion (described in detail in the vitamin C infusion section of methods). During these visits, participants completed brachial artery ultrasound measurements for assessment of endothelial function, graded cycle exercise tests using a metabolic cart to determine peak oxygen uptake (VO_2_peak), and echocardiography to determine cardiac function at rest (prior to saline/vitamin C infusion) and at peak exercise. Additionally, participants completed three bouts of constant workload exercise for assessment of oxygen uptake kinetics (VO_2_ kinetics).

### Saline and vitamin C infusion methods

Ascorbic acid (vitamin C solution-Ameri**c**an Regent Laboratories, Shirley, NY, USA) was prepared by the University of Colorado Health Pharmacy for infusion into an antecubital vein using a Harvard infusion pump [21–23]. The concentration of the vitamin C solution prepared by pharmacy was 0.06 g vitamin C per kg fat-free mass per 100 ml of normal saline. Participants received a bolus of 100 ml vitamin C solution given at 5 ml/min over 20 min followed by a “drip-infusion” given at 1.7 ml/min until vascular and exercise testing was completed or until the total dose of vitamin C administered (bolus + drip) equaled 7.5 g. Plasma vitamin C concentration was measured at baseline, after the vitamin C solution bolus, and at the end of the infusion. An equal volume of saline was infused during the saline visit to control for plasma volume.

### Blood collection and preparation

Blood was drawn via standard venipuncture technique for the measurement of glucose, insulin, HbA1C, total cholesterol, LDL cholesterol, HDL cholesterol, triglyceride, glycerol and free fatty acid concentrations. Parameters were assayed according to previously reported methods [2, 3].

### Graded exercise test

Peak oxygen uptake was determined via graded exercise to exhaustion as previously described [2–4, 6, 7] using a stationary cycle ergometer (Excalibur, Medical Graphics Corp., St. Paul, MN, USA) and a metabolic cart (Medgraphics CPX/D, Medical Graphics Corp., St. Paul, MN, USA). After the start of exercise, the work rate was increased in 10-20 W/min increments (depending on age and sex) in order to allow each participant to reach peak capacity within 7–12 min. During incremental exercise testing, the highest VO_2_ and heart rate averaged over 20 s were defined as the peak values.

### Constant work rate (CWR) exercise testing

Participants performed three identical exercise transitions from rest to CWR exercise (30 watts) on a cycle ergometer as previously described [2–4, 7–9]. Each transition consisted of a resting period to obtain baseline gas exchange data, followed by 6 min of CWR exercise. Transitions were separated by a minimum of 10 min of rest. Respiratory gas exchange measurements and heart rate data were recorded throughout each CWR bout.

### Oxygen uptake kinetic methods

Gas exchange and heart rate data for kinetic analysis were processed using a software program developed in our laboratory as previously described [10]. The data for each exercise transition were time interpolated to 1-s intervals. The three CWR exercise transitions were then time-aligned and averaged to provide a single, averaged exercise response for each subject. Pulmonary VO_2_ kinetic responses were evaluated using a 2-component exponential model allowing individual components of the VO_2_ kinetic response to be evaluated as previously reported [2, 7].

### Endothelial function

Ultrasound measurements of brachial artery FMD were performed as previously described in detail by our laboratory [24, 25] following published guidelines for assessing FMD in human participants [26]. Briefly, a pediatric blood pressure cuff was placed on the upper forearm and brachial artery images were acquired < 3–6 cm above the antecubital fossa at baseline and following reactive hyperemia produced by inflating the cuff to 250 mmHg of pressure for 5 min. After the release of the arterial occlusion, the initial 10 Doppler blood flow velocity waveform envelopes were acquired and B-mode ultrasound brachial artery diameter images were measured continuously for 2 min with the use of a GE Vivid 7 ultrasound system and a 10.0-mHz linear-array transducer as previously reported in our laboratory [4, 24] (GE vivid 7 Dimension, Milwaukee, WI, USA). All images were coded by number, blinded to group and testing condition, and analyzed by the same individual. Baseline brachial artery diameters before occlusion, the absolute change in brachial artery diameter, and the percent increase in brachial artery diameter following the release of the cuff are reported.

### Cardiac echocardiography

Two-dimensional and Doppler echocardiography [27] (GE Vivid 7 Dimension, Milwaukee, WI, USA) were performed using standard methods at rest and immediately following completion of the cycle graded exercise tests. A cardiologist blinded to the treatment allocation of the participants supervised the acquisition of these echocardiographic data and performed all of the measurements and interpretation. Participants were examined in the left lateral decubitus position using standard parasternal, short-axis, and apical views. All recordings and measurements were obtained by the same observer according to the recommendations of the American Society of Echocardiography and were be performed at the same time of day for each subject to avoid the possible influence of circadian rhythm on left ventricular diastolic function.

### Plethysmography measurements

Forearm blood flow at rest and in response to hyperemia was determined in seated participants by venous occlusion strain gauge plethysmography (D.E. Hokanson Inc. Issaquah, WA, USA), using calibrated mercury-in-silastic strain gauges and expressed as ml/100 ml/min as previously reported by our laboratory and others [4, 28]. Reactive hyperemia was calculated by subtracting baseline blood flow from peak blood flow measured following cuff occlusion.

### Statistical analysis

General linear mixed models were utilized to assess the differences in all study variables measured with saline (baseline) and vitamin C. The predictors were intervention, disease status, and the interaction between intervention and disease status; the model was also adjusted for sex. Starting from the model with the two-way interaction terms, we examined and removed the two-way interactions at an alpha = 0.05 level. All of the main effects were included in the final model. Residual diagnostics were used to assess the assumptions of our modeling approach.

## Results

### Participant characteristics and circulating factors

The presence or absence of T2D was confirmed by history including HbA1c values in all participants during the enrollment screening procedures. Baseline physical characteristics and circulating metabolic factors from research participants are presented in Table 1. There were no differences in age, body mass index, or resting blood pressure between the groups. As expected, the participants with T2D had a blood profile suggestive of diabetes compared with the healthy control group. Plasma vitamin C concentration increased with the bolus infusion of vitamin C and remained elevated throughout the trial (*P* < 0.001; controls: baseline: 72.7 ± 4.4, post-bolus: 1856.4 ± 311.0, end of trial: 985.5 ± 86 μmol/L vs. T2D: baseline: 68.3 ± 5.6, post-bolus: 1513.5 ± 82.6, end of trial: 854.2 ± 47 μmol/L). The change in vitamin C concentration achieved was not different between groups (*P* = 0.12).

### Peak oxygen uptake

Peak oxygen uptake was lower in the participants with T2D than controls at all time points in the study (Table 2; *P* < 0.01), and was not increased by acute vitamin C infusion (*P* = 0.33). RER was greater than 1.1 in all participants with all measures of VO_2_peak, consistent with maximal effort. Systolic and diastolic blood pressure (Table 2) measured at rest and at peak exercise were not different by group (*P* > 0.3) nor were these measures affected by vitamin C (*P* > 0.3). As to be expected, there was an effect of exercise on both systolic and diastolic blood pressure (*P* < 0.001 for both groups).

**Table 2:** Peak exercise capacity, oxygen kinetics, and blood pressure

	Control		T2D	
	Baseline	Vitamin C	Baseline	Vitamin C
VO_2_peak (ml/kg/min)	24.6 ± 1.4	24.9 ± 1.5	22.0 ± 0.7^†^	21.6 ± 0.8^†^
VO_2_peak (ml/min)	2244 ± 139	2267 ± 150	2129 ± 105^†^	2111 ± 108^†^
RERpeak	1.16 ± 0.02	1.16 ± 0.02	1.12 ± 0.01	1.11 ± 0.01
HRpeak	171 ± 3	171 ± 3	161 ± 4^†^	160 ± 4^†^
VO_2_ kinetics tau2 (s)	36.1 ± 3.5	35.9 ± 3.1	48.1 ± 3.8^†^	45.9 ± 3.5^†^
Systolic BP (rest)	123 ± 2	122 ± 2	119 ± 2	117 ± 2
Systolic BP (peak exercise)	193 ± 4^#^	185 ± 5^#^	188 ± 5^#^	190 ± 5^#^
Diastolic BP (rest)	81 ± 2	85 ± 2	80 ± 1	78 ± 2
Diastolic BP (peak exercise)	90 ± 3^#^	86 ± 3^#^	87 ± 2^#^	89 ± 2^#^

Data are mean ± SEM

^†^Different from control (*P* < 0.03)

^#^Different from rest (*P* < 0.001)

### Oxygen uptake kinetics

Participants with T2D had slowed VO_2_ kinetics compared with the healthy controls at baseline (Table 2; *P* = 0.03). Vitamin C infusion did not alter VO_2_ kinetics (*P* = 0.43) compared with saline infusion at baseline. The VO_2_ kinetics response to vitamin C was not different between the two groups (*P* = 0.79 for the statistical interaction of group × intervention, i.e. the response to vitamin C was not different between the groups).

### Endothelial function

**Fig. 2:**
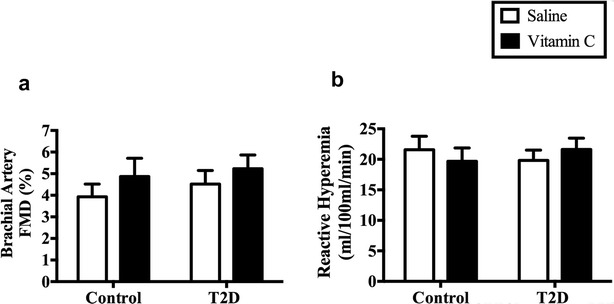
Vascular function saline and during an acute vitamin C infusion. **a** Brachial artery flow mediated dilation (FMD). **b** Reactive hyperemia in forearm

Baseline brachial artery diameter during the saline (control condition) infusion was not different between groups (Table 3; *P* > 0.2) nor were they affected by the vitamin C infusion (Table 3; *P* > 0.3). There was no difference in brachial artery FMD, either expressed as % change (Fig. 2a) or absolute change [mm; Table 3)] during the saline infusion between the healthy participants and those with T2D (*P* > 0.3). Brachial artery FMD (expressed as either % or absolute change in mm) did not change with the acute vitamin C infusion in either group (*P* = 0.09). The response to vitamin C was also not different between the two groups (*P* = 0.87 for the statistical interaction of group × intervention).

**Table 3:** Brachial artery diameter: baseline and absolute change post-cuff occlusion

	Control		T2D	
	Saline	Vitamin C	Saline	Vitamin C
Baseline diameter (mm)	4.19 ± 0.24	4.13 ± 0.24	4.32 ± 0.16	4.38 ± 0.17
Absolute change (mm)	0.21 ± 0.03	0.25 ± 0.04	0.19 ± 0.02	0.18 ± 0.02

Data are mean ± SEM

### Reactive hyperemia

Forearm blood flow was not different between the two groups during the saline infusion (*P* = 0.54). Acute vitamin C infusion did not change forearm blood flow in either group (Fig. 2b; *P* = 0.92). Response to vitamin C was not different between the two groups (*P* = 0.37 for the statistical interaction of group × intervention).

### Echocardiography

Echocardiography parameters at rest, prior to infusion of saline/vitamin C, are presented in Table 4. Lateral and septal E:E’ were elevated and septal E’ was lower in all participants prior to vitamin C infusion compared with the baseline visit pre-saline measurements (*P* < 0.04), however, all values were within normal limits. At rest, prior to saline/vitamin C infusion, controls had better cardiac contractility as measured by higher absolute circumferential strain values compared with people with T2D at all visits (effect of disease: *P* = 0.04).

**Table 4:** Resting echocardiography values

	Control		T2D	
	Saline	Vitamin C	Saline	Vitamin C
Lateral E’_rest_	0.109 ± 0.005	0.117 ± 0.007	0.115 ± 0.005	0.111 ± 0.004
Lateral E:E’_rest_	6.53 ± 0.45	7.76 ± 0.65*	6.51 ± 0.25	8.62 ± 0.38*
Septal E’_rest_	0.102 ± 0.005	0.098 ± 0.005*	0.094 ± 0.003	0.092 ± 0.003*
Septal E:E’_rest_	7.05 ± 0.64	7.76 ± 0.65*	7.95 ± 0.35	8.62 ± 0.38*
MV E:A_rest_	1.44 ± 0.13	1.37 ± 0.09	1.20 ± 0.06	1.30 ± 0.07
MV DecT_rest_ (s)	240 ± 10	238 ± 12	228 ± 11	219 ± 7
Ejection fraction (%)	65 ± 2	67 ± 2	65 ± 1	63 ± 1
Circ. strain	− 24 ± 1	− 23 ± 1	− 20 ± 1^†^	− 21 ± 1^†^

Data are mean ± SEM

*MV* mitral valve, *Circ* circumferential

* Different from saline (*P* < 0.04)

^†^Different from control (*P* < 0.03)

#### Diastolic cardiac measures

**Fig. 3:**
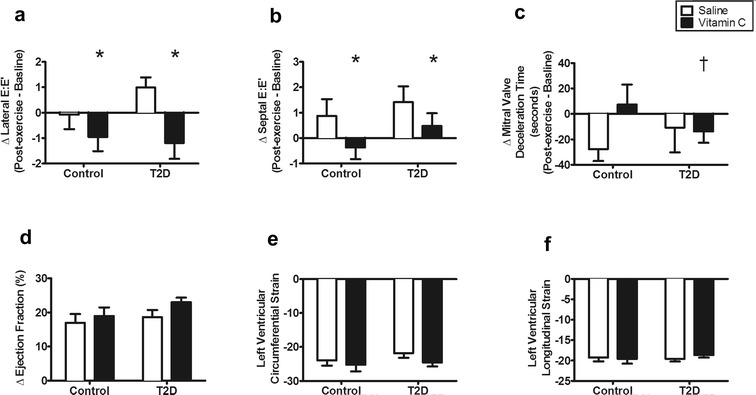
The effect of acute, peak exercise on systolic and diastolic function saline and during an acute vitamin C infusion. Data in panels **a**–**d** are presented as the change (Δ) from rest/pre-infusion to peak exercise. **a** Change in lateral E:E’. **b** Change in septal E:E’. **c** Change in mitral valve deceleration time. **d** Change in ejection fraction from rest/pre-infusion to peak exercise. **e** Left ventricular circumferential strain measured at peak exercise. **f** Left ventricular longitudinal strain measured at peak exercise. *Significantly different from saline (*P* < 0.01). ^†^Significant interaction between the vitamin C intervention and group (*P* = 0.018); mitral valve deceleration time decreased in participants with T2D with vitamin C. There was a suggestive change in ejection fraction with acute, peak exercise during vitamin C infusion (*P* = 0.057). There was a suggestive decrease in the peak exercise circumferential strain with during vitamin C infusion (*P* = 0.052)

The impact of acute vitamin C on diastolic cardiac reserve was determined by calculating the change in each diastolic parameter measured at rest and at the completion of the acute, peak exercise (graded cycle exercise test). Lateral E:E’ in response to acute, peak exercise decreased (improved) with vitamin C compared with baseline (Fig. 3a; *P* < 0.001). There were no differences in the response of lateral E:E’ to acute, peak exercise between groups (*P* = 0.56).

There was not a significant difference in lateral E’ reserve between baseline and acute vitamin C (*P* = 0.89; controls: baseline: 0.04 ± 0.01, vitamin C: 0.03 ± 0.01 vs. T2D: baseline: 0.03 ± 0.01, vitamin C: 0.04 ± 0.01). There were no differences in Lateral E’ reserve between groups (*P* = 0.58).

Septal E:E’ in response to acute, peak exercise decreased (improved) with vitamin C compared with baseline (Fig. 3b; *P* < 0.01). There were no differences in the response of septal E:E’ to acute, peak exercise between groups (*P* = 0.57).

Septal E’ reserve was greater with acute vitamin C compared with baseline (*P* = 0.03) in all participants, however, this response was greater in the control group compared with the T2D group (*P* = 0.03) (controls: baseline: 0.02 ± 0.01, vitamin C: 0.04 ± 0.01 vs. T2D: baseline: 0.02 ± 0.01, vitamin C: 0.03 ± 0.01). There were no differences in the response of septal E’ to acute, peak exercise between groups (*P* = 0.17).

Mitral valve deceleration time with peak exercise did not change with acute vitamin C infusion (Fig. 3c; *P* = 0.2). There was a significant interaction between the vitamin C intervention and group for the change in mitral valve deceleration time with acute, peak exercise (*P* = 0.02). Mitral valve deceleration time decreased (improved) in participants with T2D with infusion of vitamin C.

Acute vitamin C infusion did not alter the change in mitral valve E:A ratio with peak exercise (*P* = 0.39; controls: baseline: − 0.45 ± 0.14, vitamin C: − 0.50 ± 0.14 vs. T2D: baseline: − 0.16 ± 0.08, vitamin C: − 0.21 ± 0.06). There was no difference in the change in mitral valve E:A ratio between groups (*P* = 0.12).

In summary, the diastolic cardiac measures lateral E:E’ and septal E:E’ improved with vitamin C in both groups while mitral valve deceleration improved only in participants with T2D.

#### Systolic cardiac measures

Increases suggestive of improvement in ejection fraction with acute, peak exercise occurred during vitamin C infusion (Fig. 3d; *P* = 0.057) in both groups. These increases were not different between the groups (*P* = 0.36). There was no difference in post-peak exercise circumferential strain in the participants with T2D compared with healthy controls (Fig. 3e; *P* = 0.9). There was a suggestive decrease (improvement) in the post-peak exercise measurement of circumferential strain with acute vitamin C infusion (*P* = 0.052) in all participants. There was no difference in post-peak exercise longitudinal strain in the participants with T2D compared with healthy controls (Fig. 3f; *P* = 0.8). Additionally, there was no effect of vitamin C infusion (*P* = 0.97) on post-peak exercise longitudinal strain.

## Discussion

The primary findings of the present study were that in all participants, acute vitamin C infusion improved diastolic function in combination with statistically suggestive augmentation of systolic function but did not change FMD, forearm reactive hyperemia, or peak exercise capacity. Unique to participants with T2D, acute vitamin C infusion improved mitral valve deceleration time, a measure of diastolic function. We hypothesized that vitamin C infusion would improve cardiorespiratory fitness, and while our data did not support that hypothesis, we found other beneficial effects of acute vitamin C infusion on cardiac function in people with T2D.

Participants with T2D had lower exercise capacity and slower VO_2_ kinetics than non-diabetic controls. These findings, in addition to poorer cardiac function indicated by greater circumferential strain of the left ventricle compared with nondiabetic participants in the present study, are in agreement with our previous studies [2, 10, 12, 24]. Whole-body oxygen uptake during aerobic exercise is largely dependent on cardiac output, such that cardiac function is a primary contributor to cardiorespiratory fitness. One potential hypothesis of the mechanism of cardiovascular dysfunction in people with T2D is increased oxidative stress in T2D compared to controls [29]. Oxidative stress impairs both cardiac and vascular function through several mechanisms (reviewed previously [30]), and, pertinent to the current investigation, oxidative stress is elevated in people with T2D compared with controls [31]. Specific to our findings of improved diastolic function with acute vitamin C, excessive reactive oxygen species (oxidative stress) decrease available nitric oxide [32, 33]. In cardiac myocytes, a decrease in nitric oxide disrupts calcium kinetics [33] and impairs cardiac relaxation [34]. Previous investigations have also demonstrated improved cardiac function with acute [16] and chronic [13–15] antioxidant therapy administration. Thus, it is possible that the beneficial effect of vitamin C on diastolic function in this study was through modulation of oxidant burden. Further studies should continue to evaluate the role of vitamin C on cardiac function.

The improvement in cardiac function we observed did not correlate with an improvement in exercise capacity; these findings are similar to a recent report from our group [24]. Administering a glucagon-like peptide-1 receptor agonist over 3 months improved cardiac and vascular function in people with T2D without improving exercise capacity. The changes in cardiac function with glucagon-like peptide-1 receptor agonist, in combination with our current findings with acute vitamin C, suggest that the T2D associated exercise impairment in people with uncomplicated, well controlled T2D, could be related to skeletal muscle oxidative function, which has been reported by our group and others [2, 7, 35, 36]. Alternatively, skeletal muscle dysfunction may be the result of long-term cardiovascular impairments often observed in T2D.

Our group has previously demonstrated that a system specific approach to increase exercise capacity can be successful. In people with T2D, pharmacologically augmenting insulin sensitivity with a thiazolidinedione improved exercise capacity [3]. Improvement in insulin sensitivity likely altered multiple systems such as cardiovascular function and blood flow as well as systemic substrate uptake and utilization. Our data in rodents treated with a pharmaceutical agent that potentiates circulating glucagon-like peptide-1 by preventing its degradation, demonstrated no change in running distance with the agent alone, but did show augmentation of the exercise training response and improved cardiovascular function [37].

There are additional issues pertaining to the study design that warrant further discussion. It is not clear why the acute vitamin C infusion in the present study did not have an impact on vascular function as shown in our prior work [4] and previous investigations of others [17–19, 25]. Possible explanations include a mismatch in the timing of the effect of vitamin C on endothelial function and/or plasma concentration of vitamin C was too great, and therefore potentially pro-oxidant, to beneficially impact endothelial function in this population of participants. Conversely, the vascular measures of the participants with T2D were not different from those without T2D at baseline, which may reflect the rigid inclusion criteria for the participants with T2D. We specifically enrolled T2D subjects without clinically detectable cardiovascular disease who may have intact endothelial function. Further, measures of leg blood flow and/or skeletal muscle blood flow distribution may have provided insight regarding the link between improvements in cardiac function with the downstream impact (or lack thereof) on skeletal muscle function. Additional studies are pursuing these lines of investigation. Specifically, we recently reported a dissociation between local and global skeletal muscle oxygen extraction and VO_2_peak in people with T2D [12]. In the current study we did not assess the impact of vitamin C on this endpoint.

This study represents results from a relatively small group of patients. However, a priori power calculations based on our prior studies were used to provide sample sizes and results were robust [3, 6]. In addition, the current investigation was not powered to determine sex-specific responses to vitamin C or exercise training. Consideration of sex-specific responses in future experimental designs could provide valuable insight regarding intersection of sex and T2D on exercise capacity and cardiovascular disease risk.

## Conclusion

Cardiorespiratory fitness is a powerful predictor of all-cause and cardiovascular mortality. The link between cardiorespiratory fitness and premature death is likely strong because of the coordination of physiological systems necessary to conduct whole body aerobic exercise which makes it an appealing outcome to target therapeutically in a population at risk for premature death such as people with T2D. We sought to determine the role of cardiovascular dysfunction in diabetes-associated impaired exercise capacity. Vitamin C infusion improved cardiac function but did not alter exercise capacity. Future studies designed to generate a more complete understanding of how the physiological causes of exercise impairment in T2D may help us design optimal therapeutic approaches to treating the increased risk of premature mortality in this population.

## Abbreviations

T2D: type 2 diabetes
VO_2_peak: peak oxygen uptake
FMD: flow mediated dilation
RH: reactive hyperemia
UCSOM: University of Colorado School of Medicine
LoPAR: low-level physical activity recall
DEXA: dual-energy X-ray absorptiometry
CWR: constant work rate
